# Plasmonically Enhanced Colloidal Quantum Dot/Graphene Doped Polymer Random Lasers

**DOI:** 10.3390/ma15062213

**Published:** 2022-03-17

**Authors:** Mingxuan Cao, Min Wang, Zhiwen Wang, Luhao Zang, Hao Liu, Shuping Xiao, Matthew M. F. Yuen, Ying Wang, Yating Zhang, Jianquan Yao

**Affiliations:** 1 Department of Intelligent Manufacturing, Wuyi University, Jiangmen 529020, China; wangmin15527560331@163.com (M.W.); wangzhiwen@jiaquanip.com (Z.W.); zangluhao@163.com (L.Z.); xiaoshuping1026@163.com (S.X.); ywang@optton.com (Y.W.); 2 Key & Core Technology Innovation Institute of The Greater Bay Area, Guangzhou 510535, China; 3 Department of Mechanical Engineering, Hong Kong University of Science and Technology, Hong Kong 999077, China; meymf@ust.hk; 4 Institute of Laser and Opto-Electronics, College of Precision Instruments and Opto-Electronics Engineering, Tianjin University, Tianjin 300072, China; yating@tju.edu.cn (Y.Z.); jqyao@tju.edu.cn (J.Y.)

**Keywords:** colloidal quantum dot, random laser, graphene, surface plasmon resonance

## Abstract

An improvement in random lasers based on a colloidal quantum dot (QD)/graphene-doped polymer was observed and attributed to multiple light-scattering and graphene surface plasmon resonance. The emission characteristics of quantum dots doped with graphene oxide and reduced graphene oxide were compared. The QD/reduced graphene oxide hybrid exhibited a lower laser emission threshold (~460 μJ/cm^2^). The emission modes and thresholds were strongly dependent on both the graphene doping concentration and the external temperature. Decreased plasmon coupling was the primary reason for lower QD/graphene laser emission with increasing temperature. The optimum reduced graphene oxide concentration was 0.2 wt.%. This work provides a practical approach to optimizing the threshold and stability of random laser devices, with potential applications in displays, sensors, and anti-counterfeiting labels.

## 1. Introduction

In contrast to conventional lasers that require an optical resonator with two highly reflective mirrors to confine the photons, random lasers are cavity-free and can be achieved by extended or localized modes via multi-scattering in a disordered gain medium [1,2,3]. Random lasers have attracted considerable attention in the last few years because of their distinctive structure and mechanism, and their potential applications in mechanical devices, thermal sensors, lighting sources, anti-fraudulent labeling, speckle-free imaging, and bio-medicine [4,5,6,7,8].

Mechanisms and structures of random lasers have been extensively studied, including Anderson light localization to glassy-light behavior and the interplay between homogeneously dispersed plasmonic nanoparticles [9,10,11,12]. Graphene is a single layer, or just a few layers, of graphite that has outstanding electronic, mechanical, and optical characteristics, as well as thermal and chemical stability [13,14]. It has tremendous application potential for sensors, artificial skin, and nano-piezoelectric devices [15,16,17]. Several reports have demonstrated the feasibility of graphene-based random lasers. Marini et al. investigated the optical properties of randomly oriented undoped graphene flakes embedded in externally pumped amplifying media [18]. Ardakani et al. observed directionality and the enhancement of intensity in random lasers from a thin liquid dye layer containing graphene quantum dots (GQDs) [19].

The wrinkled structures within graphene and its porous vertical network induce multi-scattering of light emitted from the gain material and confine the photons to enable stimulated emission [20,21]. In addition, the continuous sp^2^ bonding of graphene can be interrupted by chemical oxidation [22]. The embedded sp^2^ clusters within the graphene oxide could serve as surface plasmon (SP) interaction centers and induce random laser activity [23,24]. Improved amplified spontaneous emission and random lasing from a composite structure of ZnO nanocrystals and graphene nanosheets were observed [25,26]. The underlying mechanism of the enhanced PL intensity and the random lasing could be attributed to the resonant coupling between graphene surface plasmons and an optical transition in the gain medium. The above research showed that graphene can effectively reduce the threshold of random lasers. However, these reports used plasma-enhanced chemical vapor deposition and radio-frequency magnetron sputtering, which involved complex processes, long manufacturing periods, and high costs. In addition, graphene combined with active materials via spin-coating is hard to realize in industrial applications because of poor long-term stability.

Here, ZnCdSeS/ZnS alloy quantum dots and graphene were simultaneously dispersed in a prepolymer monomer that was subsequently polymerized. After the ultraviolet curing process, the homogeneous quantum dot/graphene-doped polymer was pumped with a nanosecond laser. The random lasing thresholds and PL intensities from the samples were measured with respect to the morphologies of graphene and its derivatives. The coupling effect of graphene-based plasmons and colloidal quantum dots was discussed. We also addressed the temperature effects on the resonance characteristics of the random lasers. The emission modes and thresholds were strongly dependent on both the graphene-doping concentration and the external temperature. This work presented a simple method for fabricating a low threshold random laser from polymer doped with quantum dots and graphene. Because of its good stability, short production period, and versatility, it could be developed into a prototype for random fiber lasers, sensors, anti-counterfeit labels, and displays.

## 2. Materials and Methods

**Figure 1:**
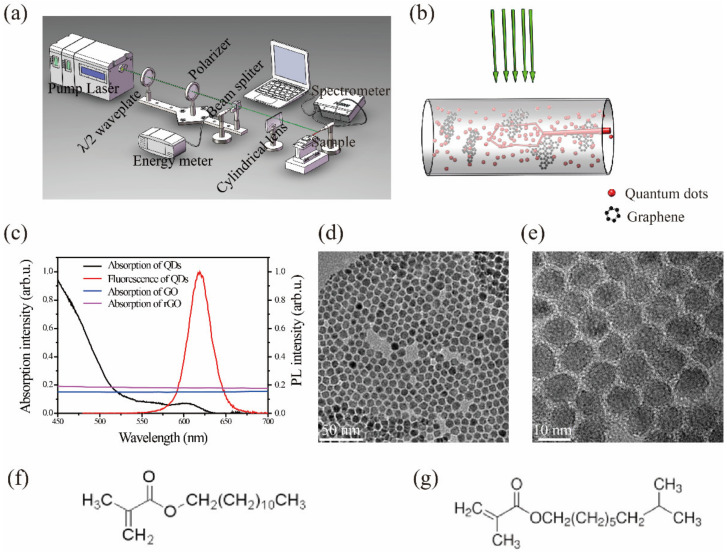
(**a**) Experimental setup for acquiring emission spectra of the samples, (**b**) schematic of the multiple light scattering within the samples, (**c**) absorption and PL spectra of samples, (**d**,**e**) transmission electron microscope images of ZnCdSeS/ZnS quantum dots, (**f**,**g**) monomer structures before polymerization.

ZnCdSeS/ZnS quantum dots were prepared as reported previously [27]. Figure 1f,g show the typical monomer structures before polymerization, and the mass ratio of the two monomers is 1:1. The polymer matrix of all samples were polymerized from the above monomers. Figure 1c shows the absorption and photoluminescence (PL) spectra of the QDs. The emission peak was at 630 nm, with a 28 nm full width at half maximum (FWHM). The QDs exhibited good crystallinity and homogeneity with an average size of 10 nm, as imaged via transmission electron microscopy (Figure 1d,e). The QDs (20 wt.% in the monomer) and graphite oxide (0.1 wt.%, Jiangsu XFNANO Materials Tech., 0.5–5 μm lateral sizes) were added to the acrylic resin monomer. The mixture was stirred for 3 h. The solution was then sonicated in an ice bath for 4 h to exfoliate the graphite oxide into graphene oxide (GO) sheets (labeled as QD-GO). The ice bath avoided further reactions of the graphene oxide. To ensure the effects of surface functional groups on the graphene, the QDs (20 wt.%) and reduced graphene oxide (rGO, 0.1 wt.%, Jiangsu XFNANO Materials Tech., 0.5–5 μm diameter) were added to the monomer (labeled as QD-rGO). After doping the rGO, the mixtures were stirred at room temperature for 3 h in the dark for homogeneous mixing. QD-doped monomers were also prepared as a reference (labeled as bare QD). All of the samples were filled into capillary tubes and rapidly cured for 2 min with an ultraviolet lamp (3 mW/cm^2^).

Figure 1a,b show the experimental setup for random laser measurements and determining the QD/graphene mechanism. The capillary was fixed on a three-dimensional translation stage and pumped by the 532 nm second harmonic of a Q-switched Nd: YAG laser (10 ns,10 Hz). The excitation beam was focused on the capillary tube by a cylindrical lens. The pump area was fixed at 5 mm ×1 mm. A fiber optic spectrometer (HR-2000, 0.02 nm resolution) collected the laser emission spectrum along the capillary direction. UV-vis absorption and PL spectra were measured by UV-1780, Shimadzu, Kyoto Japan and HR-4000, Ocean Optics, Largo, FL, US, fluorescence spectrometers, respectively. The QD morphology was imaged with a transmission electron microscope (JEM-2100F, JEOL, Tokyo, Japan). A D8 Advance X-ray diffractometer (D2 PHASER, Bruker, Bremen, Germany) was used to acquire the samples’ X-ray diffraction (XRD) patterns.

## 3. Results and Discussion

### 3.1. Graphite Oxide Exfoliated into GO

**Figure 2:**
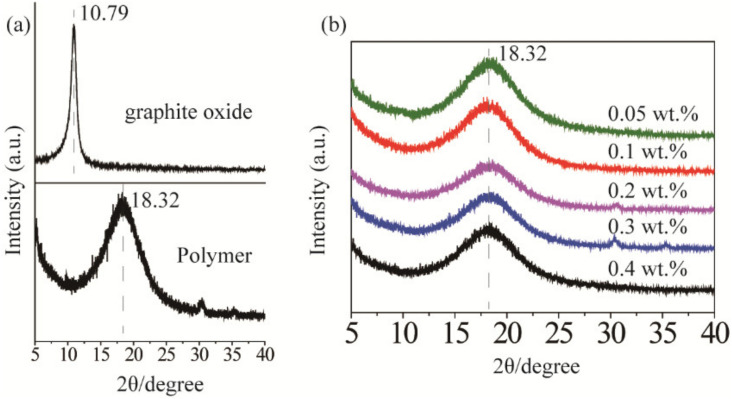
(**a**) X-ray diffraction (XRD) patterns of graphite oxide and polymer, (**b**) XRD patterns of graphene oxide at different concentrations in the polymer.

XRD characterization was used to indirectly analyze the graphene exfoliation and dispersion in the polymer matrix. As shown in Figure 2a, two characteristic diffraction peaks at 10.79° and 18.32° corresponded to the stacked structure of the graphite oxide layer and the polymer matrix [28]. After ultrasonic treatment, the diffraction peaks of the graphite oxide flakes disappeared (see Figure 2b), which indicated that they were exfoliated into GO and dispersed in the polymer matrix. As shown in Figure 1c, the absorption spectrum of GO and rGO overlapped with the fluorescence spectrum of QDs (580 nm~640 nm), ensuring that the graphene network could confine the light through SPR, and leading to an enhanced emission [26,29]. However, the light absorption of graphene can cause a strong fluorescence quenching. Therefore, there should be an optimal range for the doping amount of GO and rGO.

**Figure 3:**
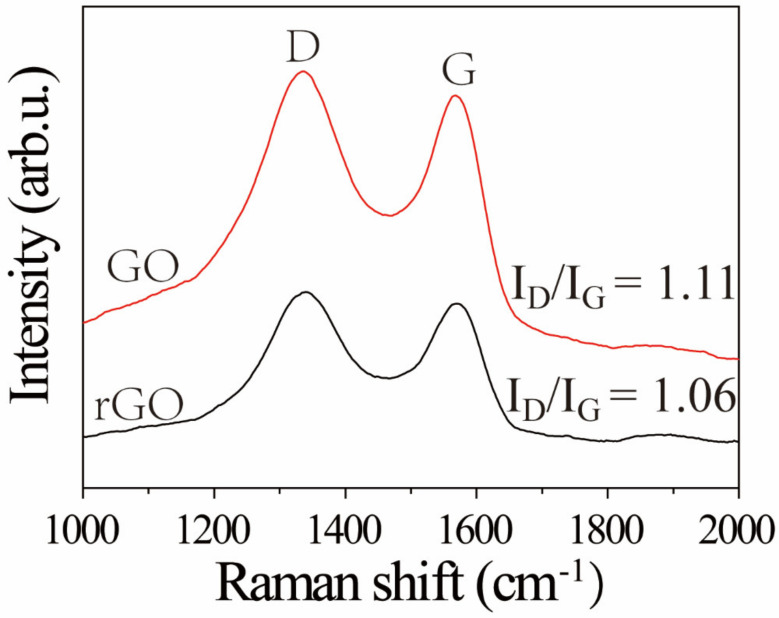
Raman spectra of GO and rGO powders.

Raman spectra of GO and rGO were measured by a Raman spectrometer with laser of 532 nm excitation. As displayed in Figure 3, the GO and rGO samples showed two peaks at 1580 and 1350 cm^−1^ corresponding to G and D bands. The G band was generated from the vibration of sp^2^-bonded. The D band was generally correlated with defects derived from vacancies, grain boundaries, and amorphous carbon species [30]. The I_D_/I_G_ ratio of the GO was lower than that of rGO, indicating that the number of smaller graphene domains increased [31].

### 3.2. Random Laser from Different Graphene Derivatives

**Figure 4:**
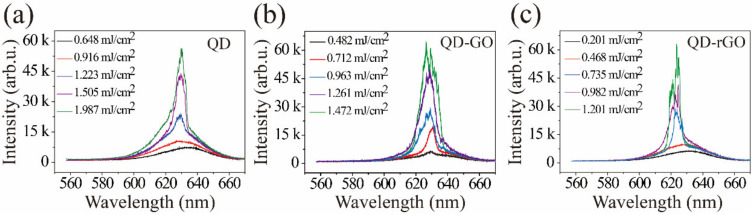
(**a**) Random laser emission spectra of bare QD, (**b**) QD-graphene oxide, and (**c**) QD-reduced-graphene oxide.

Figure 4 depicts random laser emission spectra of bare QD, QD-GO, and QD-rGO for various pump powers ranging over 0.2–2.1 mJ/cm^2^. No sharp peaks were observed at low pump energies. Above the laser threshold, the QD-GO and QD-rGO samples exhibited coherent random-lasing and numerous sharp emission peaks appeared. The FWHMs of spikes were less than 0.1 nm (see in Table 1). These phenomena indicated that multiple micro-resonators were randomly formed inside the QD-GO and QD-rGO, and each resonator had a specific emission wavelength. Depending on the composition of the gain medium and the scattering medium, random lasers can be divided into devices with non-distributed and distributed feedback [8]. In the present experiment, the gain medium and the scattering medium in these composite structures were uniformly mixed, which is characteristic of random lasers with disordered distribution feedback. Due to the lack of necessary conditions (no scattering medium) for the formation of the microresonator, the sample of bare QD exhibited incoherent random lasing with no sub-nm spikes.

Figure 4b shows random laser emission spectra of the QD-GO sample; its emission threshold was lower than those for the bare QD samples. After the graphite oxide was ultrasonically exfoliated into graphene oxide, the specific surface area significantly increased, the photon mean-free path was reduced, the probability of forming a closed optical circuit increased, and the emission threshold was lowered. The emission threshold of the QD-rGO sample was lower than that of the QD-GO sample, as shown in Figure 4c. The difference in morphologies and distributions of QD-GO and QD-rGO is one reason for the difference in the lasing threshold. In addition, carbon materials contain a large number of sp^2^ and sp^3^ bonds [32]. It has been experimentally demonstrated that the size of initially present sp^2^ domains did not increase with GO reduction. Instead, the reduction usually creates small sp^2^ clusters, which are smaller in size, but numerous. In turn, these sp^2^ clusters create isolated molecular states that eventually percolate to mediate the transport of carriers via hopping, which enhances the plasmon density [33]. Surface plasmonic resonances (SPR), excited by the light interaction with the graphene surface plasma, can increase the QD spontaneous emission rate and enhance light emission. As reported previously [26], coupling of the SPs in the underlying graphene strongly reduced the lasing threshold. The plasmonic density of states and the SP-QD coupling efficiency strongly depended on the graphene’s number and types of functional surface groups. Here, the lower lasing threshold of the QD-rGO related to that of the QD-GO was consistent with previous reports [34].

### 3.3. Temperature Test

**Figure 5:**
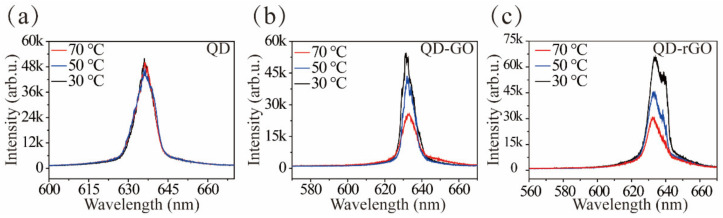
(**a**) The effect of temperature on random laser emission spectra of bare QD, (**b**) QD-GO, and (**c**) QD-rGO.

Surface plasmon resonances is temperature-dependent, as reported previously [34,35]. Figure 5 depicts changes in the PL spectra of the QD, QD-GO, and QD-rGO samples as a function of temperature. The emission intensity of the bare QD changed little with increased temperature, as shown in Figure 5a. However, the emission spectra of QD-GO and QD-rGO decreased significantly with temperature, as shown in Figure 5b,c, and sharp peaks decreased in intensity as well. The plasmon-coupling is expected to decrease at high temperatures due to the reduction of the plasmon density of states and the enhancement of plasmon damping caused by electron-phonon interactions, thereby decreasing the PL enhancement ratio [25]. Moreover, the changes in the lasing mode may be ascribed to the deformation of the graphene, which has a negative thermal expansion coefficient [36]. This would lead to the structural change in a QD/graphene mixture with rising working temperature.

### 3.4. Random Laser Test of rGO Doped with Different Mass Ratios in CQD

**Figure 6:**
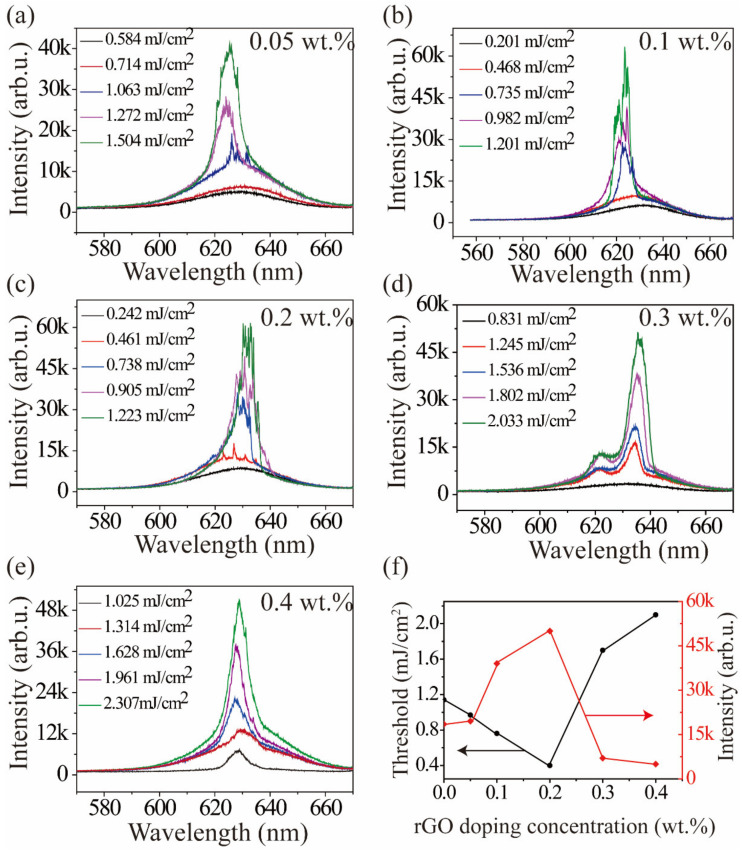
(**a**–**e**) Random laser emission characteristics of reduced graphene oxide at different concentrations; (**f**) the peak output intensity (red line) and laser threshold (black line) of colloidal quantum dots as a function of reduced graphene oxide concentrations.

The effect of rGO concentration on the emission characteristics of QD-rGO samples was examined for additional rGO doping at 0.05 wt.%, 0.1 wt.%, 0.2 wt.%, 0.3 wt.%, and 0.4 wt.%, as shown in Figure 6. Because the generation of a random laser is dependent on the scattering to form closed-loop paths, it can be concluded that different concentrations of graphene doping had different scattering mean-free lengths and exhibited different lasing actions. A low graphene concentration showed weak feedback of backscattered light, and the photon mean-free path was large, reducing scattering and the probability of forming a closed-loop cavity. Thus, the laser emission threshold was relatively high in Figure 6a,b. When the rGO density in the polymer was too high, the penetration depth of the 532 nm pump light significantly decreased, and the effective pump volume in the active medium could be too small to provide sufficient optical gain, as shown in Figure 6d,e. Hence, QD-rGO with a 0.2 wt.% doping concentration had the best laser characteristics (Figure 6c), with the lowest laser emission threshold (460 μJ/cm^2^), as shown in Figure 6f.

## 4. Conclusions

We demonstrated improved random laser activity using a polymer doped with colloidal quantum dots and graphene. In this system, graphene was the scattering medium and supplied SPR coupling with the lasing emission. We could conclude from the temperature-dependence experiments that reduced SP coupling was the primary cause for decreasing QD-graphene laser emission with increased temperature. The plasmon density of states and the SP-QD coupling efficiency strongly depended on the graphene’s number and types of surface functional groups; hence, QD-rGO had a lower laser emission threshold (~460 μJ/cm^2^). The optimum doping concentration of rGO in the polymer was 0.2 wt.%. Overall, this work provides a practical approach for optimizing the threshold and stability of random laser devices, with potential applications in sensors and confidentiality tags.

## Figures and Tables

**Table 1:** Random laser emission characteristics of different samples.

Sample	Threshold (mJ/cm^2^)	FWHM (nm)
Bare QD	1.14	8.50
QD-GO	0.79	0.10
QD-rGO	0.65	0.06

## Data Availability

Not applicable.
